# Gaze Tracking Based on Concatenating Spatial-Temporal Features

**DOI:** 10.3390/s22020545

**Published:** 2022-01-11

**Authors:** Bor-Jiunn Hwang, Hui-Hui Chen, Chaur-Heh Hsieh, Deng-Yu Huang

**Affiliations:** 1 Department of Computer and Communication Engineering, Ming Chuan University, Taoyuan 333, Taiwan; bjhwang@mail.mcu.edu.tw (B.-J.H.); 09166071@me.mcu.edu.tw (D.-Y.H.); 2 College of Artificial Intelligence, Yango University, Fuzhou 350015, China; chxie@ygu.edu.cn

**Keywords:** gaze tracking, deep learning, convolutional neural network (CNN), long short-term memory (LSTM)

## Abstract

Based on experimental observations, there is a correlation between time and consecutive gaze positions in visual behaviors. Previous studies on gaze point estimation usually use images as the input for model trainings without taking into account the sequence relationship between image data. In addition to the spatial features, the temporal features are considered to improve the accuracy in this paper by using videos instead of images as the input data. To be able to capture spatial and temporal features at the same time, the convolutional neural network (CNN) and long short-term memory (LSTM) network are introduced to build a training model. In this way, CNN is used to extract the spatial features, and LSTM correlates temporal features. This paper presents a CNN Concatenating LSTM network (CCLN) that concatenates spatial and temporal features to improve the performance of gaze estimation in the case of time-series videos as the input training data. In addition, the proposed model can be optimized by exploring the numbers of LSTM layers, the influence of batch normalization (BN) and global average pooling layer (GAP) on CCLN. It is generally believed that larger amounts of training data will lead to better models. To provide data for training and prediction, we propose a method for constructing datasets of video for gaze point estimation. The issues are studied, including the effectiveness of different commonly used general models and the impact of transfer learning. Through exhaustive evaluation, it has been proved that the proposed method achieves a better prediction accuracy than the existing CNN-based methods. Finally, 93.1% of the best model and 92.6% of the general model MobileNet are obtained.

## 1. Introduction

Most people rely on vision in receiving information. Fixation is the main way to receive visual information. Observing eye movements can observe the cognitive process and concentration [1]. Therefore, tracking the estimated gaze is considered as the most effective way to evaluate visual behavior. The technology of gaze tracking has been gradually applied in different fields in recent years, such as Human–Computer Interaction (HCI), audience gaze behavior analysis, advertising analysis, gaze information collection and analysis on webpages, medical disease interpretation training [2,3].

In recent years, due to the development of machine learning technology, many studies have used deep learning training methods to improve the accuracy of gaze point estimation [4,5]. Since the Convolutional Neural Network (CNN) excels in many computer vision tasks, it is also used for gaze point estimation [2,6,7]. For CNN-based gaze point estimation, the image is selected as the training and predictive data with the extracted spatial features. Deep learning requires a lot of data for training to avoid overfitting. There have been several publicly available datasets for gaze estimation [8,9,10,11,12,13]. Many training samples for gaze estimations are mainly image-based [8,9,10]. Few samples are video-based collected from people viewing dynamic stimuli such as watching YouTube and Netflix [11,12,13].

In terms of appearance-based gaze estimation, the authors of [8] constructed a LeNet-inspired CNN to learn the mapping between 2D head angle, eye image and output gaze angle. Another study [9] constructed a dataset containing 64,000 appearance-based images from 50 subjects. In a controlled environment where the subject’s head was fixed, images of the subject were collected from a fixed distance. These images possessed dense sampling of gaze angles. Krafka et al. [10] used GazeCapture to obtain a large amount of gaze data using mobile phones as vehicles, and used these data as training data for their network, iTracker, a convolutional neural network for eye tracking. In terms of providing video datasets, the datasets of [11] contained images recorded by color and depth cameras with head pose of the participant, visual target, and recording conditions. However, the dataset only recorded the data of three subjects under two different environmental conditions. The Discrete screen target drew a small circle evenly every 1.1 s at a random position on the screen. The Continuous screen target moved the circle along a random trajectory for 2 s. In [9,11], although all the above datasets captured a large number of head poses and appearances, the experiments were conducted in a strictly controlled manner without changes in body postures. A strictly controlled experimental environment was not a practical live condition in which a subject’s head and body moved naturally influenced by the emotional changes during exploring media contents. The research reported in [12] collected the gaze point, head position and direction in the three-dimensional space as the data when asking participants to look at the predetermined target on the monitor. The test targets were arranged as 3 × 3 and 4 × 4 fixed points on the screen. The participants were instructed to gaze from the fixed point to the center or from the center point to the target point. The study of [14] estimated the gaze on a tablet. The goal was to perform an uncalibrated gaze estimation with the front camera of the tablet, where the posture and method of holding the tablet were not limited. In [12,13], both articles traced the gazed area of interest and gaze patterns with small circles as the test target object, regardless of static graphics or dynamic movies.

In our previous research, three types of fed images and varied network architectures are proposed to estimate the gaze point and compare the efficiency on the architecture of applying the different convolutional layers, using batch normalization (BN) and applying the global average pooling layer (GAP) instead of the fully connected layer [15]. The input model of the face image is the best output result. Furthermore, there is a correlation between time and consecutive gaze positions in visual behaviors when people watch a dynamic material (such as a movie). Usually, the consecutive gaze positions are not too far apart. Therefore, this paper proposes to use the exploration results as the basis for the gaze point estimation design. In addition to the spatial feature, the temporal features are considered to improve the accuracy. The traditional neural network models for gaze estimation use a single image as the input training data but rarely use videos. If the video can be used for predicting, the effect can be better achieved on the gaze estimation through the context of the time series in the video.

Unlike traditional neural networks of which input data are independent from output data, a time-related machine learning network such as the Recurrent Neural Network (RNN) uses the output of the previous step as the input of the current step. However, the performance of RNN in long-term memory is not as expected, and Long Short-Term Memory (LSTM) is designed to improve the defect of the long-term memory [16,17]. LSTM was proposed by Hochreiter and Schmidhuber [16] in 1997 to solve the gradient descent problem of standard RNN. Since the standard RNN has only a simple structure, it is difficult to capture long-term memories. Therefore, LSTM introduces three gates that control memory in the neural unit to control the retention of memory: Forget Gate, Input Gate, and Output Gate.

LSTM has been applied in many fields, such as language modeling, text classification, activity recognition, acoustic modeling and stock market prediction, etc. LSTM is more commonly used early in stock returns prediction [18]. There are also studies using LSTM to explore time-sequential features in videos. For example, there are some studies using CNN and LSTM to propose some methods for action recognition [19,20,21,22,23,24]. The gaze-based early prediction of the user’s intention is proposed by employing a combination of CNN and LSTM [25]. By exploring literature studies, it rarely can be found combing both extracted spatial and temporal features at the same time for gaze estimation. For the research topic on gaze estimation, the available features for the training samples are mostly the extractions of eyes and face images [15]. Based on the above exploration, this research article hence proposes to concatenate spatial and temporal features and has named it as CCLN (CNN Concatenating LSTM Network) to improve the performance of gaze estimation in the case of time-series videos as the input training data.

Many research works focus on network architecture to improve performance or reduce complexity, such as GAP and BN [26,27]. In order to reduce the parameters of convolutional neural networks and avoid overfitting, the GAP layer is proposed to replace the fully connected layer [26]. The difference between the GAP layer and the fully connected layer is that the average value of each feature map of the last layer is taken out. Ioffe et al. [27] proposed the method of batch normalization to overcome the problem of hard-to-train models. The main idea is by making normalization a part of the model architecture and performing the normalization for each training mini-batch. The benefits of BN include improving the learning speed, reducing the dependence on initial parameters, and eliminating the need for dropout layer in some cases. Therefore, in this paper, the proposed model can be optimized by exploring the influence of GAP and BN on CCLN. 

There are a few research works on gaze estimation that use videos based on temporal features as the input training data. This research article evaluates the performance of different network architectures and hyperparameter adjustments considering both spatial and the temporal features. Conventionally, prediction accuracy is often used as a metric to evaluate classifiers. However, the complexity of the model is also a very important design factor, because it will affect the calculation load and implementation cost of the system. Thus, in this research, several prediction metrics are used, including network complexity, accuracy, loss, recall, F1 score and precision for the evaluation and comparison of the networks. The major contributions of this work are summarized as follows:

- In addition to model architecture, it is generally believed that larger amounts of training data will lead to better models. We propose a method for constructing a video dataset for gaze point estimation based on concatenating spatial and temporal futures.
- We propose a simple, yet effective CNN Concatenate LSTM network (CCLN), to explore the performance of gaze estimation in the case of time-series videos as the input training data. Through exhaustive evaluation, we prove that the proposed method is achieving better prediction accuracy than the existing CNN-based methods.
- This research takes account of the studied effect of the batch normalization and global average pooling methods into the designed network on better prediction accuracy and reducing complexity.
- The effectiveness of different commonly used general models and the impact of transfer learning are studied.

The remainder of this paper is organized as follows. Section 2 first gives an overview of the proposed method, describes the data pre-processing algorithm, and then, the design of CCLN. The numerical analysis and performance comparison are given in Section 3. Finally, the conclusion is drawn in Section 4.

## 2. Proposed Concatenating Spatial and Temporal Features Method

In this section, first, an overview of the proposed concatenating spatial and temporal features method is stated, showing and explaining the system architecture. Second, the descriptions on the data pre-processing, including synchronizing data collection, labeling and building dataset are addressed. Last, the design of CCLN consisting of three functional blocks, CNN Block, LSTM Block and Output Block, is presented.

### 2.1. System Overview

**Figure 1:**
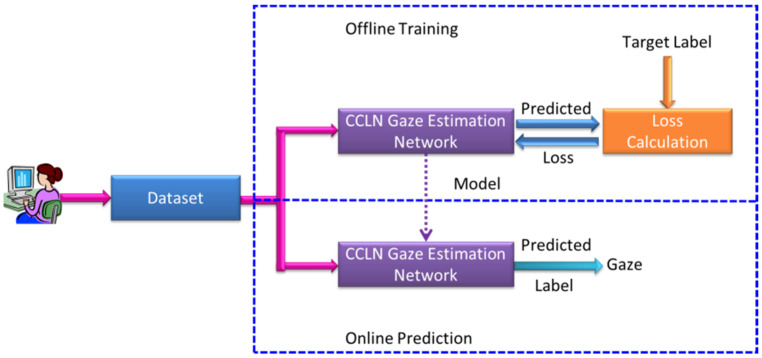
System architecture.

Figure 1 shows the system architecture for gaze point estimation based on concatenating spatial and temporal futures, including Offline Training and Online Prediction procedures. There are three function blocks consisting of the proposed Dataset, CCLN Gaze Estimation Network, and Loss Calculation. In the Online Prediction procedure, the prediction model is transferred when the Offline Training procedure is terminated. In our previous research [15], the input model of the face image was the best output result; therefore, this study continues to use such type of images as the training and prediction data.

In the past, video was rarely used as the input data for gaze point estimation. This paper proposes to record facial images while participants watching a movie, supplemented by the gaze point coordinates labelled by the eye tracker, to create a dataset with time-series-synchronized data. This dataset will be used as the training sample of the CCLN proposed in this paper. CCLN Gaze Estimation Network is designed for concatenating spatial and temporal features. In the prediction procedure, video (i.e., Stimulus video) is used as an estimation unit. 

This paper mainly completes several tasks including the following:

1. Synchronized data collection and automatic labeling method

Both the quantity and the content of training data are important factors that affect the final performance of machine learning. Data labeling is a heavy task. Designing an automatic labeling method can speed up and increase the construction of a training dataset.

2. Constructing a dataset by watching movies

There are some public datasets built for viewing moving objects, mainly known objects such as small circles. They made participants gaze on the small circle on the screen, and then mark the connection coordinating this known object and the eye or face image. However, the visual behavior occurred in this kind of experimental environment setting is very different from that of generally watching a movie. There are few datasets that use videos (face) as the training data, so it is necessary to build a suitable time-series-related dataset. In this paper, the proposed method is to obtain the images taken by a webcam when participants viewing each movie frame. Further labeling the image to coordinate with the gaze point estimated by the infrared eye tracker to build a dataset of the synchronized time-series. However, such a taken image is still too messy and not suitable for direct use as a training sample, so the image needs to be cropped to a fixed size. In addition, a method is proposed to compose the taken images into a video with the consideration of an appropriate length.

3. Designing the network architecture of CCLN

Other than using the eye image as the main extraction feature for gaze estimation, using the face image can overcome the influence caused by the head movement. Therefore, the training of CCLN will take the built video dataset as the training sample with the face image as the input image. Moreover, a complicated network architecture will easily lead to overfitting when the input image consists of few features. Hence, the number of LSTM layers, batch normalization and global average pooling are used to explore a model to obtain a balance between better prediction accuracy and reducing complexity. The performance, including accuracy, loss, recall, F1 score and precision, etc., are computed.

4. Studying the performance of commonly used models and the impact of transfer learning

This paper evaluates the performance of CNN when it is replaced by commonly used network models, including VGG16, VGG19, ResNet50, DenseNet121 and MobileNet. In addition, whether or not the effect of trained weights is initially used during training is evaluated, as a way to explore the generality of the network architecture proposed in this paper.

### 2.2. Synchronizing Data Collection, Labeling and Building Dataset

In order to obtain datasets of eye and face images for labeling, it is common to manage the viewable screen area into M × N regions with showing a predetermined target to be viewed, the target usually as a circle appearing in the center or moving toward the center of each region area in a random manner [12,13]. The eye images and the face images tracing the target to be viewed are recorded and marked during the viewing process. There are two problems with the abovementioned obtainment of the datasets. Firstly, the data obtained are limited to the center position of gazed region blocks, and the amount of data is relatively insufficient. 

**Figure 2:**
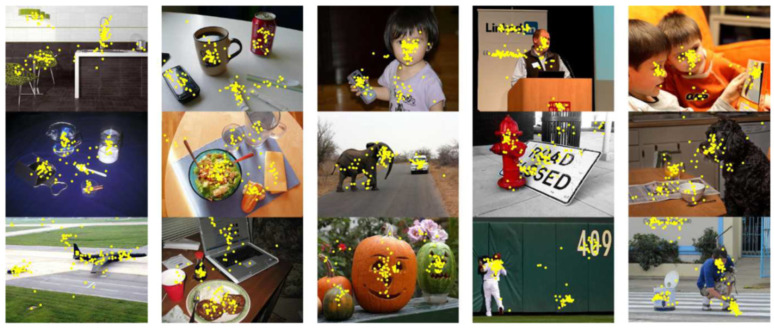
Most gaze points are distributed near the center of the interested object [28].

Secondly, by observing the results of static images [28,29], which have been marked with the gaze points, it can be found that the gaze points are actually concentrated on the objects of interest. For example, it can be proved from Figure 2 [28] that the position of the object in the image will affect the visual behavior, which will affect the area of interest for viewing, so the abovementioned obtainment of the datasets [12,13] is not favorable. Besides, although datasets such as [28,29] contain gaze point information, they lack the eye image at this time and cannot be directly used for training. 

**Figure 3:**
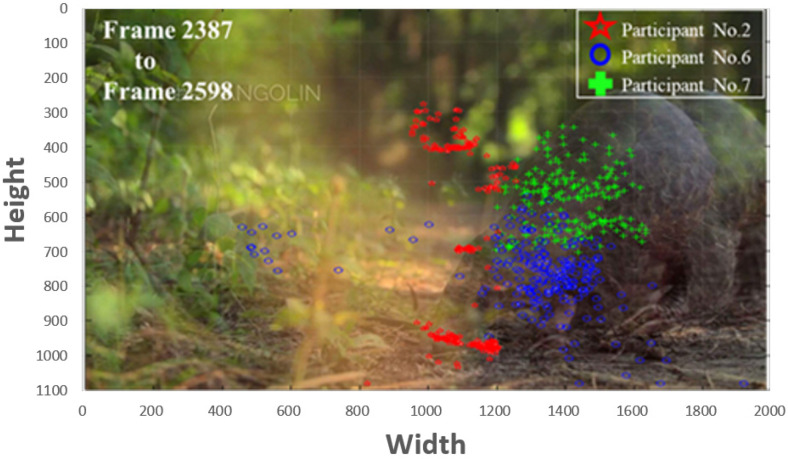
Comparing the gaze points of the different participant during frame 2387 to frame 2598.

In addition, we have conducted an experiment on the gaze behavior of the participants while watching the arranged video regarding to the pangolin object. Figure 3 shows the examples that the visual behavior exhibits personal preferences with distinctly different distributions of gazes. It exhibits the result of comparing the gaze points of the different participants during frame 2387 to frame 2598 to express the attentions. Comparing to participant No. 2, the distributions of gaze points of participants No. 6 and No. 7 are more aggregated. Even though the areas of interest are different, all of the three participants have attended the pangolin object. This result also demonstrates that the gaze point is clustered and time-related.

For obtaining the training dataset, this research paper proposes to adopt an eye tracker to record gaze points of the participants while watching the video of general films. There are currently some eye trackers that use infrared light to detect gaze points with acceptable accuracy, and they have been adopted by some studies [28]. For the eye tracker to achieve higher detection accuracy, a calibration process needs to be conducted prior to engaging the participant in the recording process initiated by the eye tracker. One should be also aware that the functionable distance for an eye tracker to detect gazes is relatively short as well as sever head and body movements might have impacts on detection accuracy. This infrared type of eye tracker is an acceptable auxiliary tool for acquiring gaze points to implement the proposed automatic labeling. Furthermore, a webcam along with the eye tracker is used for acquiring face images at the meantime to build the corresponding eye and face datasets. 

To achieve the automatically labeling data for training, the acquired data of gaze points detected by the eye tracker and face images captured by the webcam must first be synchronized, in terms of the timestamps and the corresponding frame numbers. Therefore, this research paper designs a method for obtaining the synchronized data. In order to keep simultaneously with the frame rate of the video, the webcam is set to capture the face image of the participant while watching the video with one shot per frame. That is, the frequency of taking face images is the same as the frequency of the video frame rate. In the meantime, the (x, y) coordinates and time (frame number) of the current gaze point taken by eye tracker are recorded. The synchronization is implemented by using the frame number as the unit of time to associate the data between the coordinates of a gaze point and a captured face image. In order to achieve the purpose of automatically labeling data, in the program development, we first play the video and record the number of the frame, then capture the face image and get the gaze point detected by the gaze tracker at this time. Finally, the number of the frame, gaze point and face image serial number are combined into a record of data for storage. When training the input video, the corresponding relevant annotation data can be obtained at the same time.

Furthermore, the face images obtained by the webcam need to be cropped prior to be used as the training samples. Generally, facial recognition using facial landmarks for calibrating is to adjust the face to the center of the picture, the eyes of the face are on the horizontal line, and the face size of each picture is scaled to the same size. This study uses facial landmarks for facial recognition and using the center of the frame as the alignment base to crop the obtained face images. Since the heads and bodies of participants might move in pitch, roll and yaw with the distance changes from the camera or participants posse different eye heights, there are different image patterns of face and eye when participants look at the same position. These variations of different image patterns corresponding to the same gaze point should be regarded as visual behavior characteristics. If the obtained face image is cropped with the face as the alignment center, part of the features will be lost. To retain these features, the obtained face image will be cropped relative to the center of the frame.

It is observed that when gaze points gather on a certain object of interest in a short time, there will be no large-scale movement of adjacent positions. When the object of interest is changed, gaze points may shift away to another interested object with more movements. Based on the above observation on gathering effects and distinguished movements of shifting gaze points associated to the object of interests, this study proposes a method to composite the captured corresponding face images into the video as the training dataset. Gaze is dynamic and the probability of having consecutive gaze points with the same coordinates is extremely low. The face images are encoded into video to build the dataset with the following rule. This study presents the concept of a B × B macroblock, with size of width B pixels and height B pixels, as a basis for composing the training data. If the nth frame is the first frame of the video to be produced, we calculate the distance of the gaze points between the nth frame and the (n + 1)th frame. If the distance is within B/2 pixels, we record this frame and continue to calculate the next frame until it exceeds B/2 pixels, and then proceed to the next calculation procedure. The face images mapping to frames in the same recording are converted into a video, i.e., a training or test data in the dataset. The face images of the gaze points in a macroblock of B × B pixels are converted into a training data. The lengths of the composed training videos will adversely affect the training results. The built dataset is randomly split into 0.7, 0.2, and 0.1 for training, testing, and evaluation sets, respectively. A built dataset is a labelled folder storing data collected from each participant, respectively. Prior to performing training and testing the model, data in a respective dataset folder are randomly selected and split into 7:2:1 for training, testing, and evaluation sets. The approach of splitting the datasets into training and testing datasets can be reproducible.

The experimental tools for collecting data and 6 selected videos to watch are the same as used in [15], including a 22-inch with 16:9 screen is used as the video playback device, Logitech C920r HD Pro Webcam and Tobii Eye Tracker 4C, respectively, for capturing face image and getting the gaze point. The participants watched 6 selected videos, each with a size of 1920 × 1080 pixels and a frame rate of 24 frames per second, played in a randomly selected manner. The detailed information of the videos is shown in Table 1. The experiment and data are newly conducted in accordance to this research purpose. In this study experiment, there are 28 participants and face images of them are collected. However, the data type used in this paper is different. The input data use video instead of images, whereas [15] used images.

**Figure 4:**
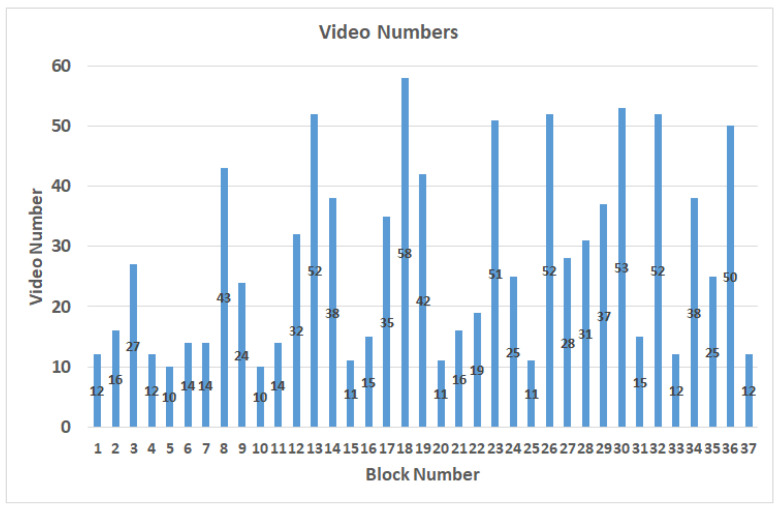
The distribution results after labeled as blocks.

**Figure 5:**
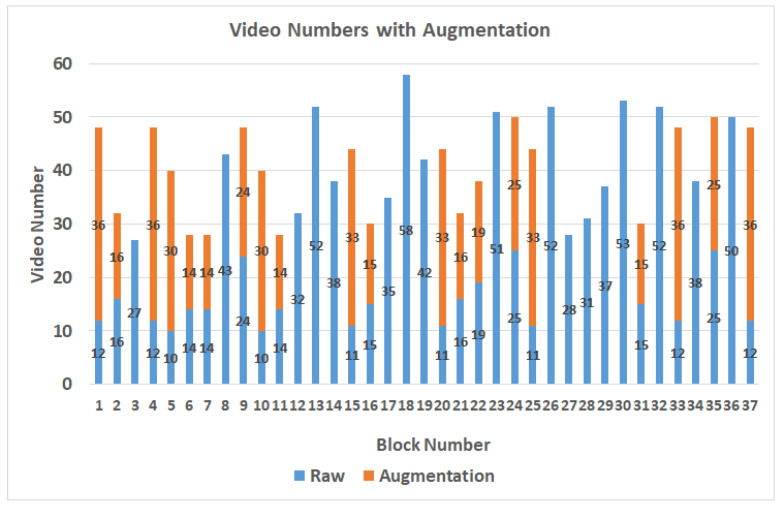
The distribution results after data augmentation.

In the case of setting B to 30, after the production of the video, the video length distribution is less than 5 s, and 90% is within 1 s. Therefore, the threshold is set as 3 s, and if it exceeds, it will be divided into 2 equal segments. The dataset contains 1017 videos labeled with block numbers out of the 37 blocks proposed by [15]. Figure 4 shows the distribution results after the videos are labeled as blocks. According to the proposed video splitting method, the length of each video is controlled within 3 s and the distribution is shown in Table 2. When the participant is watching the video, the gaze points are usually in the area near the center of the screen, resulting in an uneven data distribution. The data augmentation method of brightness adjustments will be performed to expand the number of videos in blocks which contain fewer videos than the average number of videos in all blocks. Thus, the blocks with a lower number of videos can get enough data, and finally, there are 1517 videos after data augmentation, as shown in Figure 5.

### 2.3. The Proposed CCLN Gaze Estimation Network

**Figure 6:**
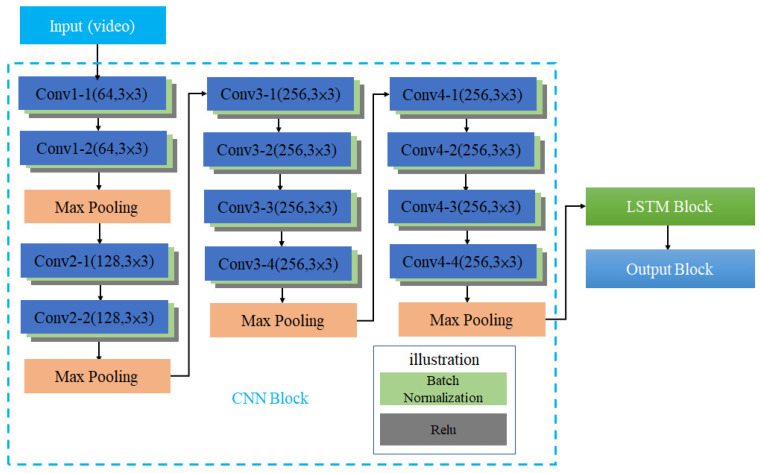
The architecture of CCLN Gaze Estimation Network.

Figure 6 shows the architecture of CCLN Gaze Estimation Network consisted of 3 functional blocks, CNN Block, LSTM Block and Output Block. The architecture of CNN Block includes 12 layers of convolutional layers and 4 layers of Max Pooling. The number of convolutional layers is connected in series with 2, 2, 4, 4, and the middle is connected with the Max Pooling layer. After each convolutional layer in the model, batch normalization (BN) and Relu functions are added to reduce the computational complexity and are illustrated as light green and gray, respectively. The architecture of this CNN block is similar to that of [15]. The difference is that there are no global average pooling (GAP) and Softmax layers, so it can have a better effect on transfer learning. The dataset built in this paper is randomly split into training, testing, and evaluation sets. According to the system architecture shown in Figure 1, in the Offline Training state, a video in the training set is randomly selected and decoded into frames to input CCN in CCLN for training. For each built dataset, data collected from per participant, respectively, data in a respective dataset folder are randomly selected and split into 7:2:1 for training, testing, and evaluation sets. During performing Offline Training state, a video will be randomly selected from the training set, and then, the next one will be randomly selected, so forth and so on. Data for performing testing and evaluation are as well randomly selected with the same approach. In addition, the technique of early stopping is applied to end the training procedure. Then, the testing set is used to validate the performance of the trained model. Finally, the evaluation set is used to evaluate the effectiveness of the trained model including accuracy, loss, F1 score, recall and precision in the Online Prediction state. The LSTM Block analyzes the system performance including the following different conditions: the number of LSTM layers, the comparison of unidirectional and bidirectional LSTM architectures, and whether to use Dropout, BN. Usually, the Output Block includes fully connected (FC) and Softmax layers, the issue of the impact of replacing fully connected layer with GAP will be evaluated and presented in Section 3.1.2. 

## 3. Numerical Analysis

In this work, we evaluate the performance for CCLN Gaze Estimation Network including layers of LSTM, unidirectional and bidirectional LSTM, to use Dropout and BN, GAP. In addition, the comparison of various models is presented.

### 3.1. The Evaluation for LSTM Block

#### 3.1.1. Layers of LSTM, Directional LSTM

The multi-layer LSTM network is used to extract temporal features layer by layer and improves the robustness of the model [30]. That the multi-layer LSTM can be stacked and temporally concatenated to form more complex structures has been applied to solve the real-life sequence modeling problem [31]. Several researchers made use of the bidirectional LSTM to improve the performance [32,33]. The bidirectional LSTM is the most performant to LSTM and GRU classifiers for fall event detection [32]. In [33], they apply bidirectional LSTM for real-time anomaly detection in surveillance networks. Therefore, in this paper, we study the effects of various architecture issues on the design of networks including bidirectional LSTM and number of layers in estimation the gaze network. Table 3 shows the types of LSTM Block architectures to be evaluated.

- Type 1 unidirectional

Compared with [15], the accuracy of proposed CCLN is higher than [15] in the face scheme as shown in Table 4, with an approximately 4% performance improvement. The results show that using video as the input training data and using network of CNN concatenating LSTM can improve the accuracy for gaze estimation by 3.36%.

B. Type 2 bidirectional

Compared with the unidirectional LSTM architecture, the bidirectional LSTM enables the model to make more accurate classifications as shown in Table 5, with an approximately 2.69% performance improvement.

C. Type 3 unidirectional multi-layers

**Figure 7:**
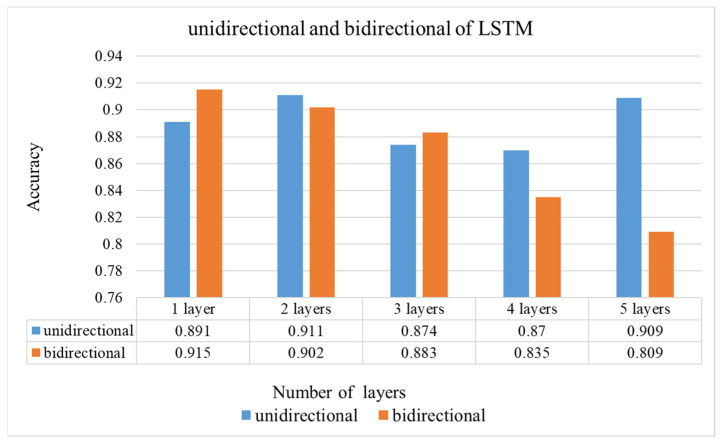
The accuracy of multiple layers for different LSTM architecture.

The next experiment is to compare the results of multiple layers for the unidirectional LSTM architecture as shown in Figure 7. When the number of layers is increased from 1 to 2, the accuracy is increased to 0.911. However, when it increases to 3 or 4, the accuracy reduces to 0.874 and 0.87, respectively. From the results, it presents that LSTM needs to use the appropriate number of layers for training. A better model is not necessary engaging in more layers.

D. Type 4 bidirectional multi-layers

According to the results of multiple layers for the unidirectional LSTM architecture as shown in to Figure 7, the accuracy using 2 layers of LSTM is higher than using 1 layer. Since the correlation on positions between gaze points is close, the accuracy of 3 layers and 4 layers shows a downward trend. The reason why bi-directional is higher than unidirectional LSTM is that for objects of interest, gaze points usually concentrate in a small area in a short time, as shown in Figure 3. Therefore, feedback information is useful to improve the accuracy. When the number of LSTM layers increases, the accuracy shows a downward trend. The possible reason is that if there are not many features, the architecture is too complicated and affects the interpretation. As it can be seen from Figure 7, 1 layer with bidirectional and 2 layers with unidirectional architectures have relatively high accuracy outcomes.

**Figure 8:**
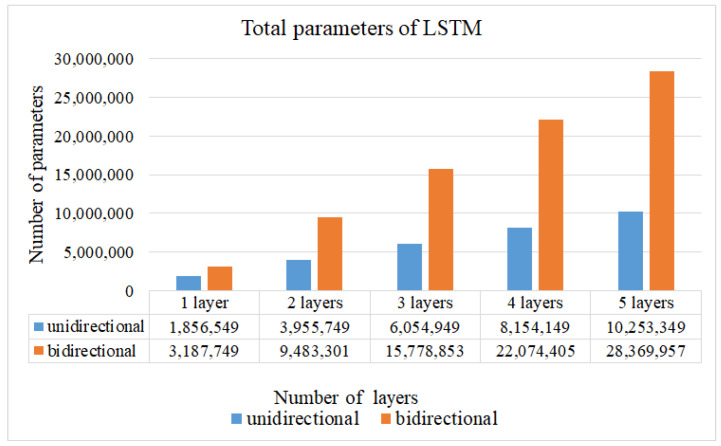
The total parameters for different layers, and unidirectional and bidirectional of LSTM.

The computational complexity of the network can be estimated by the total parameters of the network. The total parameters for different layers, and unidirectional and bidirectional of LSTM shown in Figure 8 are presented to contrast the efficiency, the more parameters the more layers of LSTM. By observing the results in Figure 7 and Figure 8, the 2-layer unidirectional LSTM has 768,000 more parameters than the 1-layer bidirectional LSTM, and the accuracy rate is also reduced by 0.4%. Therefore, the performance of the 1-layer bidirectional LSTM is used to compare with others networks [34,35].

#### 3.1.2. GAP

This subsection discusses the comparison of the results of using GAP after the LSTM. The GAP can reduce the number of parameters of the model, thus the training time can be reduced accordingly. The experimental results are shown in Table 6. The addition of GAP reduces the overall parameter amount by about 13%, and the accuracy of the model is increased by about 1.2%. In addition, it has a relatively good performance in other evaluation metrics including loss, F1 score, recall and precision. 

#### 3.1.3. Dropout and BN

The use of dropout can prevent the occurrence of overfitting and improve the generalization ability of the model. The use of BN can increase the convergence speed during learning, thereby increasing the stability of the neural network. For the evaluation of the influence of making use of dropout and BN on system performance, the overall experimental results are shown in Table 7. The results show that the accuracy 93.1% of the 1- layer LSTM with bidirectional architecture using GAP and BN is the best model. In Table 7, it shows that the performance is improved by adopting the BN method comparing to Table 6. 

### 3.2. The Comparison of Various Models

The result of Section 3.1 is based on the model of CNN in the proposed CCLN using [15]. In order to consider the utility of using the general network architecture, this study evaluates several general CNN models, including VGG16, VGG19, ResNet50, DenseNet121 and MobileNet [34,35]. Evaluating the impact of transfer learning on overall performance is as following.

#### 3.2.1. Comparison of Various General Models

To compare the five commonly used general models with pre-trained ImageNet database and a 1-layer unidirectional LSTM, the experimental results are shown in Table 8. The results show that the accuracy of 71% of the MobileNet model is the highest. In general, the four models of VGG16, VGG19, ResNet50, and DenseNet121, all increase the depth of the network by increasing the number of convolutional layers, thereby increasing the accuracy. This deeper network model is mainly used to identify 1000 categories of ImageNet; however, there are a few object features to be identified in this study since the facial images are used as the input data. 

Compared with the previous four deep CNN models as shown in Table 3, the MobileNet model has a lower accuracy by using Pre-trained ImageNet which has few convolutional layers. CCLN uses the facial images as the input data, and the features of facial images are not as complex as those used in ImageNet. Therefore, an accuracy rate of using MobileNet model with Pre-trained ImageNet is 71%, a reduction of about 77% of the total parameters can be achieved. 

#### 3.2.2. Comparison of Multi-Layers LSTM

According to Table 8, although the accuracy of MobileNet and DenseNet121 is not much different, the MobileNet has the lowest total parameter amount. The performances of MobileNet cascaded multi-layers LSTM with unidirectional or bidirectional are compared as shown in Table 9 and Table 10. Both of them have the best accuracy with 1 layer of LSTM, and the accuracy of bidirectional has increased to 91.4%, that is, it is better than unidirectional, and the result is consistent with Figure 7. From this experiment, we know that using more layers of LSTM will not make the accuracy higher whether it uses unidirectional or bidirectional. In addition, through the feedback and training of the previous and future feature sequences by the bidirectional LSTM, a better classification output can be made.

#### 3.2.3. GAP

The evaluated results of MobileNet cascaded 1-layer LSTM with bidirectional and using GAP instead of fully connected are shown in Table 11. According to the results, using GAP, the total parameter amount is reduced by about 7%, the time required for model training is shortened. In addition, the model accuracy rate is improved by 1.2%. Additionally, the performance is improved in other evaluation metrics including loss, F1 score, recall and precision.

#### 3.2.4. Dropout and BN

Using dropout and BN to evaluate MobileNet cascaded 1-layer LSTM with bidirectional and adopting GAP, the experimental results are shown in Table 12. Even if the accuracy of the model is not much different from the unused one, both are around 92%. It is known that dropout can improve the generalization ability and using BN can speed up the convergence. Therefore, the impacts of using dropout and BN to MobileNet on the performance are also evaluated. Among them, the accuracy, F1-score, recall and precision of cascading 1-layer LSTM with bidirectional and using GAP are the highest. Using dropout will cause the same results as shown in Table 7 causing a performance degradation.

#### 3.2.5. Transfer Learning

The previous experiment using general model is based on ImageNet data for pre-training. The content of ImageNet is a collection of general objects, including people features. According to the model proposed in [15], gaze estimation is trained with faces as the input data, that is, facial image input is used for pre-training to extract features. Therefore, this paper also evaluates the effect of adding face and eyes to pre-train on the performance by using the images acquired from [15], both with 8000 images, respectively. The results are shown in Table 13. 

According to the results in Table 13, it is known that whether adding faces or eyes for CNN network pre-training, the accuracy is about 89% to 92%, which is not much different from the best model obtained in the previous experiment, 93% in the Table 7. It can be observed that using the pre-trained model with added images of faces and eyes cannot outperform the best model obtained in the previous experiment. Consequently, the CCLN in this paper proposes to use video as the input data. Whether CNN uses the pre-trained model of the paper [15] or uses the general model of MobileNet as ImageNet to pre-train, the performance can be improved by 8.1% and 7.4%, respectively. The total number of parameters of the best model obtained in the previous experiment as shown in Table 7 is about 9.5% less than the pre-trained models in Table 13. 

## 4. Conclusions

This paper proposes a CCLN architecture with video as input to estimate gaze. By studying the impact of the number layers of LSTM, unidirectional or bidirectional, BN, dropout and GAP on detection accuracy to obtain optimal hyperparameters, we designed a simple, yet effective CCLN. The results show that using the CNN developed in [15] with a cascaded 1-layer LSTM with bidirectional and using GAP and BN has the highest accuracy of 93.1%. In case of adopting general model MobileNet, using ImageNet as the pre-trained model instead of CNN has the best accuracy 92.6%, as shown in Table 12. We also study the impact of transfer learning by adding face and eyes images to pre-train. The results show that it is not helpful to improve the accuracy of the model. It can be observed that whether using a CNN with a specially modified architecture or a general model widely used, the accuracy of the model can be improved for our proposed CCLN. 

In the literature, BN is widely used to improve performance. The proposed CCLN of this paper also confirms the improvement results of using BN. Using BN to improve performance also can be found in the model proposed in [15] or in general models of CNN. Nevertheless, the performance of evaluation metrics, including accuracy, loss, F1 score, recall and precision, cannot be improved by adopting dropout.

By inputting facial images with different features such as beards to evaluate the performance will be the future directions. In addition, using GAN (Generative Adversarial Network) to generate other facial features, such as the face costumes of entertainers, and to evaluate the impact introduced by different features, is an attractive issue.

## Figures and Tables

**Table 1:** The details of six animated videos [15].

Title	Resolution (Pixel)	Length (s)
Reach	1920 × 1080	193
Mr Indifferent	1920 × 1080	132
Jinxy Jenkins & Lucky Lou	1920 × 1080	193
Changing Batteries	1920 × 1080	333
Pollo	1920 × 1080	286
Pip	1920 × 1080	245

**Table 2:** The number of video lengths.

Length (s)	Amount
1	991
2	21
3	5

**Table 3:** The types of LSTM Block architectures to be evaluated.

Type	CNN	LSTM
1	CNN Block	unidirectional
2	CNN Block	bidirectional
3	CNN Block	unidirectional multi-layers
4	CNN Block	bidirectional multi-layers

**Table 4:** The accuracy compared with [15] for Type 1.

	[15]	Proposed CCLN
Accuracy	0.862	0.891

**Table 5:** The accuracies for Type 1 and Type 2.

	Type 1	Type 2
Accuracy	0.891	0.915

**Table 6:** The performance for using GAP.

	1-Layer Bidirectional LSTM	1-Layer Bidirectional LSTM + GAP
Total parameters	6,345,157	5,839,301
Accuracy	0.915	0.927
Loss	0.361	0.324
F1 score	0.915	0.929
Recall	0.919	0.935
Precision	0.916	0.929

**Table 7:** The performance for using Dropout and BN.

	1-Layer Bidirectional LSTM + GAP + Dropout	1-Layer Bidirectional LSTM + GAP + BN	1-Layer Bidirectional LSTM + GAP + Dropout + BN
Total parameters	5,839,301	5,841,349	5,841,349
Accuracy	0.918	0.931	0.926
Loss	0.302	0.359	0.351
F1 score	0.919	0.930	0.925
Recall	0.923	0.935	0.931
Precision	0.920	0.930	0.926

**Table 8:** The performance for various general models.

CNN Model	VGG16	VGG19	ResNet50	DenseNet121	MobileNet
Pre-trained	ImageNet	ImageNet	ImageNet	ImageNet	ImageNet
Total parameters	19,194,725	24,504,421	29,061,157	27,294,917	6,636,389
Accuracy	0.6	0.616	0.685	0.705	0.71
Loss	1.5	1.38	1.18	1.06	1
F1 score	0.588	0.596	0.671	0.693	0.7
Recall	0.605	0.621	0.692	0.709	0.718
Precision	0.597	0.601	0.664	0.688	0.7

**Table 9:** The performance of MobileNet [36] cascaded multi-layers LSTM with unidirectional.

LSTM Layer	1 Layer	2 Layers	3 Layers
Pre-trained	ImageNet	ImageNet	Imagenet
Total parameters	6,636,389	8,735,589	10,834,789
Accuracy	0.71	0.625	0.572
Loss	1	1.27	1.45
F1 score	0.7	0.607	0.54
Recall	0.718	0.634	0.578
Precision	0.7	0.618	0.537

**Table 10:** The performance of MobileNet [36] cascaded multi-layers LSTM with bidirectional.

LSTM Layer	1 Layer	2 Layers	3 Layers
Pre-trained	ImageNet	ImageNet	ImageNet
Total parameters	6,900,581	13,196,133	19,491,685
Accuracy	0.914	0.876	0.863
Loss	0.358	0.549	0.71
F1 score	0.913	0.879	0.865
Recall	0.92	0.878	0.868
Precision	0.914	0.889	0.87

**Table 11:** The performance of MobileNet with or without adopting GAP.

GAP	Without	With
Pre-trained	ImageNet	ImageNet
Total parameters	6,900,581	6,394,725
Accuracy	0.914	0.926
Loss	0.358	0.317
F1 score	0.913	0.926
Recall	0.92	0.929
Precision	0.914	0.928

**Table 12:** The performance of MobileNet using dropout or BN.

	Without	Dropout	BN	Dropout + BN
Pre-trained	ImageNet	ImageNet	ImageNet	ImageNet
Total parameters	6,394,725	6,394,725	6,396,773	6,396,773
Accuracy	0.926	0.92	0.923	0.922
Loss	0.317	0.305	0.407	0.287
F1 score	0.926	0.92	0.924	0.921
Recall	0.929	0.924	0.929	0.925
Precision	0.928	0.923	0.925	0.924

**Table 13:** The performance of transfer learning.

Pre-Trained	Add Face and Eyes	Add Face	Add Eyes
Total parameters	6,394,725	6,394,725	6,394,725
Accuracy	0.919	0.924	0.891
Loss	0.341	0.332	0.537
F1 score	0.915	0.922	0.813
Recall	0.918	0.921	0.875
Precision	0.911	0.924	0.883

## Data Availability

If researchers are interested in using the collected datasets, the datasets can be made available by sending request emails to the authors.
